# Effects of a glyphosate-based herbicide on soil animal trophic groups and associated ecosystem functioning in a northern agricultural field

**DOI:** 10.1038/s41598-019-44988-5

**Published:** 2019-06-12

**Authors:** Marleena Hagner, Juha Mikola, Irma Saloniemi, Kari Saikkonen, Marjo Helander

**Affiliations:** 1 Faculty of Biological and Environmental Sciences, Ecosystems and Environment Research Programme, University of Helsinki, Niemenkatu 73, 15140 Lahti, Finland; 2 Department of Biology, University of Turku, 20014 Turku, Finland; 3 Biodiversity Unit, University of Turku, 20014 Turku, Finland; 4 Plant Health, Natural Resources Institute Finland (Luke), 31600 Jokioinen, Finland

**Keywords:** Ecosystem services, Environmental impact

## Abstract

Despite an increasing concern of consequences of using vast amounts of glyphosate-based herbicides in agroecosystems, their potential effects on non-target soil organisms and soil functioning are mostly unknown. It has also been argued that fields in northern latitudes should be under special surveillance as the short active period of decomposers may restrict glyphosate degradation. We investigated the effects of a glyphosate-based herbicide, Roundup, on the abundance of enchytraeids and nematodes, both essential groups in decomposer food webs, and plant litter mass loss and soil availability of mineral N in a two-year agricultural field setting in south-west Finland. Our experiment consisted of (1) non-treated weed plots, (2) plots, where weeds were killed by hoeing, and (3) plots treated with both Roundup and hoeing. We found that killing plants by hoeing had drastic effects on soil fauna and functioning, and apparently, distinguishing these effects from direct glyphosate effects is profoundly important when evaluating glyphosate risks in soils. In contrast, the effects of Roundup on soil fauna and functioning were minor and transient and no glyphosate remains were found in the soil at the end of the experiment. These results suggest that side-effects can be minor and glyphosate degradation effective also in soil under northern climatic conditions.

## Introduction

Glyphosate, or N-(phosphonomethyl)glycine, is a broad-spectrum, nonselective and post-emergence herbicide, used as an active ingredient in several weed killing products since 1970^1^. Due to its effectiveness against wide variety of plants, glyphosate has been nominated as the once-in-a-century herbicide^2^, and currently, it is one of the most commonly used herbicide in agricultural and non-agricultural cultivation systems in developed countries. For example, in Finland glyphosate comprised 67% of all herbicide-active ingredients sold in 2016^3^. When ending up in the soil, glyphosate is quickly adsorbed to soil particles and has a low probability of leaching along with surface waters or downwards into the soil profile^4^. Glyphosate is also vulnerable to microbial degradation and its main degradation product, aminomethylphosphonic acid (AMPA), is strongly adsorbed to soil solids. For these reasons, glyphosate has generally been regarded as an environmentally safe herbicide^2,5^. Recent studies have, however, shown that the degradation and adsorption rate of glyphosate and AMPA greatly depend on soil properties^6,7^, including their phosphorus status^8^. The biosafety of glyphosate has been questioned especially in northern ecosystems, where glyphosate might persist longer because of prevalent soil types and climatic conditions^9,10^. Due to the rise of public concern, the safety of glyphosate was recently thoroughly assessed by the European Union Member States and the European Food Safety Authority (EFSA), and in December 2017, the European Commission renewed the glyphosate approval for five years^11^. This decision was, however, mostly based on experiments adhering to the guidelines of traditional toxicology tests, promulgated by pesticide-regulatory authorities, while *in-situ* experimental tests, focusing on community and ecosystem level effects, are scarce.

Soil decomposer communities, or food webs, consist of a vast diversity of interacting organisms^12,13^. These serve as the base of a healthy soil nutrient chain by breaking down dead organic material, releasing nutrients in mineral forms for plant uptake and improving soil structure and aggregation^14^. The activity of decomposers is essential to the well-being of all organisms relying on primary production, and to maintain the ecosystem services they supply over time, a few keystone groups and processes are fundamental. Bacteria and fungi, often together called microbes, are the primary decomposers due to their almost irreplaceable capability of breaking down complex organic compounds. They form the majority of decomposer biomass and are largely responsible for organic matter decomposition, nutrient transformations and degradation of toxic compounds. Their activity and nutrient mineralization are, however, regulated by soil animals, such as nematodes and enchytraeids that feed on microbes^15–18^. Because of such importance in soil functioning, these groups have been suggested as suitable bioindicators for soil health and quality^19^. Nematodes and enchytraeids also well represent soil food webs as they together cover all trophic groups of soil fauna: enchytraeids are detritivorous, feeding on dead organic matter and microbes living on that^20^, while nematodes include bacterial-feeding, fungal-feeding, root-feeding, omnivorous and predatory genera^21^. Although the link between biodiversity and processes in the soil is not straightforward due to the complexity of food webs that govern the processes^13^, changes in the structure of decomposer food webs have a potential to affect the key ecosystem services, such as litter decomposition and nutrient cycling, which they supply. Considering the widespread use of glyphosate-based herbicides in agroecosystems, their impacts on decomposer communities and processes in agricultural soils thus warrant careful consideration.

Surprisingly, despite the enormous use of glyphosate-based herbicides around the world and the active research around glyphosate products^22^, little is known of their potential effects on non-target soil organisms. The effectiveness of glyphosate is based on the inhibition of 5-enolpyruvylshikimate-3-phosphate synthase (EPSPS) in the shikimate acid pathway, which in turn interferes with the production of aromatic amino acids and secondary compounds required in the defense functions of plants and many microbes^23,24^. The shikimate pathway is present not only in plants, but also in many taxa of soil fungi and bacteria, and in principle, glyphosate effects on soil organisms could either be intimate (when organisms growing in soil or on dead plant remains are subjected to glyphosate) or mediated through modified plant chemistry in withering plants. In greenhouse studies, glyphosate application has recently been found to affect microbial composition and enzymatic activity in plant rhizospheres (i.e. in the soil adhered to plant roots) as well as in bulk soil^25–28^. Disrupting effects of glyphosate on earthworms^29^ and their interactions with symbiotic mycorrhizal fungi^30,31^ have also been reported. However, there is much controversy about the effects of glyphosate on soil microbial communities and activities^32–34^ and notably, few studies have so far been carried out in field conditions^35^. Moreover, it is typically difficult in experiments to disentangle the actual toxic effects of the herbicide on soil communities and their functioning from the inevitable effects that arise of terminating plant growth and input of carbon-rich compounds in the plant rhizosphere. As the evidence of the effects of glyphosate-based herbicides on soils *in-situ* is scarce, and so far focuses entirely on microbes and earthworms, more attention is clearly required of the potential side-effects of these compounds on other key soil organisms and their associated ecosystem services^29,30^. It has also been argued that a special focus should be paid to soils in northern countries, where the short active period of decomposition and plant growth (4–6 months) may restrict the degradation of glyphosate^9^.

In this study, we investigated the effects of the most commonly used glyphosate-based herbicide, Roundup, on the abundance of soil nematodes and enchytraeids, accompanied by the ecosystem services they contribute to, in a two-year agricultural field setting in south-west Finland. We established an experiment with three well-replicated treatments, consisting of (1) plots of non-treated Weeds (W plots), (2) plots, where the weeds were killed by Hoeing (H plots), and (3) plots, where the weeds were first killed with Roundup and which then, once the plants had withered, were Hoed to achieve similar soil structure and disturbance as in H plots (RH plots). By comparing the abundance of soil organisms and soil functioning among these three treatments we intended to disentangle the possible, direct toxic effects of Roundup (by contrasting RH and H plots) and the inevitable indirect effects caused by the destruction of live vegetation (by contrasting H and W plots). Abundances of nematodes and enchytraeids have earlier been shown to promptly respond to disturbances in vegetation and soil^36–39^ and the response of bacterial- and fungal-feeding nematodes can be used as an indicator of microbial growth^40–42^. To examine the effect of Roundup on soil ecosystem services supplied by the animals and microbes together, we further measured the mass loss of control litter and litter sprayed with Roundup and soil availability of mineral N in each treatment plot. Based on earlier findings that glyphosate is quickly adsorbed to soil particles and effectively degraded by soil microbes, we hypothesized that:

1. While the destruction of live vegetation in H plots will have effects on the abundance of soil fauna (due to decreasing live plant and increasing dead plant resources), litter mass loss (due to changes in abiotic conditions) and soil mineral N availability (due to decreasing plant N uptake and increasing N mineralization) in comparison to W plots,
2. No differences in these variables will be detected between RH and H plots.

## Results

### Soil moisture

Mean soil moisture varied from 25 to 30% among the four samplings in June and October in 2016 and 2017, but was not affected by plot treatments in either upper (F = 0.91, P = 0.438 for sampling × plot treatment interaction effect, and F = 0.74, P = 0.490 for plot treatment effect) or lower soil layer (F = 2.20, P = 0.057 and F = 1.33, P = 0.290, respectively).

### Enchytraeid biomass

**Figure 1:**
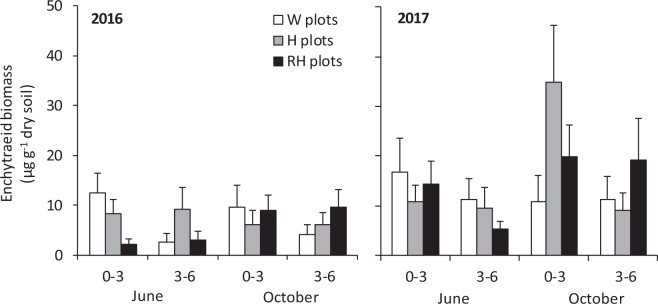
Biomass of enchytraeids (mean + s.e., n = 10) in the two layers of soil (0–3 and 3–6 cm) in field plots with non-treated weeds (W plots), hoeing (H plots) or Roundup application followed by hoeing (RH plots) in June and October samplings in 2016 and 2017. No statistically significant differences of mean biomass were found in H plot–W plot and RH plot–H plot comparisons (for the significance of soil layer and sampling month effects, see Table 1 and Supplementary Table 1).

Enchytraeid biomass was not affected by plot treatments or sampling month in either year (Fig. 1, Table 1, Supplementary Table 1). In the June 2016 sampling, the biomass was on average higher in the upper than lower soil layer, but the marginally significant soil layer × plot treatment interaction effect (P = 0.057) suggests that this difference occurred in W plots only (Fig. 1, Table 1).

**Table 1:** F and P statistics of repeated measures ANOVA of the effects of soil layer (0–3 cm or 3–6 cm, a repeated measure) and plot treatment (non-treated weeds, hoeing, Roundup application followed by hoeing) on nematode numbers and enchytraeid biomass in early (June) and late (October) growing season in 2016 and 2017.

	Soil layer		Soil layer × Plot treatment		Plot treatment	
	F	P	F	P	F	P
**June 2016**						
Enchytraeid biomass	6.28	**0.022**	3.38	0.057	2.12	0.149
Total nematode number	4.77	**0.042**	5.68	**0.012**	2.15	0.145
Bacterivores	1.20	0.288	4.92	**0.020**	3.79	**0.042**
Fungivores	1.68	0.211	4.25	**0.031**	0.81	0.462
Root feeders	1.01	0.328	1.44	0.263	1.39	0.274
Omnivores	10.4	**0.005**	0.36	0.702	0.66	0.527
Predators	12.1	**0.003**	1.42	0.269	1.15	0.340
**October 2016**						
Enchytraeid biomass	0.07	0.797	0.43	0.657	0.68	0.518
Total nematode number	0.92	0.350	0.56	0.582	1.43	0.266
Bacterivores	1.21	0.286	0.60	0.561	2.26	0.133
Fungivores	<0.01	0.950	0.38	0.690	0.58	0.572
Root feeders	8.97	**0.008**	2.47	0.113	0.50	0.615
Omnivores	5.23	**0.034**	1.90	0.179	0.48	0.628
Predators	7.93	**0.011**	1.05	0.369	1.49	0.253
**June 2017**						
Enchytraeid biomass	1.09	0.310	0.60	0.561	0.19	0.831
Total nematode number	4.38	0.051	5.87	**0.011**	35.5	**<0.001**
**October 2017**						
Enchytraeid biomass	2.75	0.114	1.50	0.249	1.16	0.337
Total nematode number	1.55	0.229	1.26	0.306	7.27	**0.005**

Field replicate block was included in ANOVA models, but is not reported in the table. Statistically significant (P < 0.05) effects are marked in bold.

### Total number of nematodes

**Figure 2:**
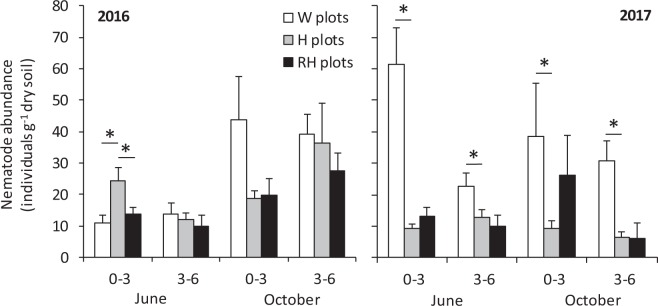
Total abundance of nematodes (mean + s.e., n = 10) in the two layers of soil (0–3 and 3–6 cm) in field plots with non-treated weeds (W plots), hoeing (H plots) or Roundup application followed by hoeing (RH plots) in June and October samplings in 2016 and 2017. Statistically significant differences of mean abundance in H plot–W plot and RH plot–H plot comparisons are depicted with a line and an asterisk (for the significance of soil layer and sampling month effects, see Table 1 and Supplementary Table 1).

In June 2016, the total number of nematodes was affected by a significant soil layer × plot treatment interaction, while no treatment effects were found in October (Fig. 2, Table 1). The interaction effect was because H plots had a higher number of nematodes than W plots and because RH plots had less nematodes than H plots in the upper soil layer (0–3 cm), but not in the lower soil layer (3–6 cm) (Fig. 2). Looking the interaction from another angle suggests that the upper soil layer had more nematodes, but in H plots only (Fig. 2). The lower soil layer had a generally higher number of nematodes in October than June, while in the upper layer this trend was found in W plots only (Fig. 2, Supplementary Table 1).

In 2017, the plot treatment effect was clear in both samplings and both soil layers: H plots had on average 75% less nematodes than W plots, whereas RH plots did not differ from H plots (Fig. 2, Table 1, Supplementary Table 1). The significant soil layer × plot treatment interaction effect in June was because the magnitude of treatment effects was greater in the upper than lower layer (Fig. 2). In contrast to 2016 samplings, the number of nematodes was not significantly affected by the sampling month in either of the two soil layers (Fig. 2, Supplementary Table 1).

### Abundance and relative proportions of nematode trophic groups

**Figure 3:**
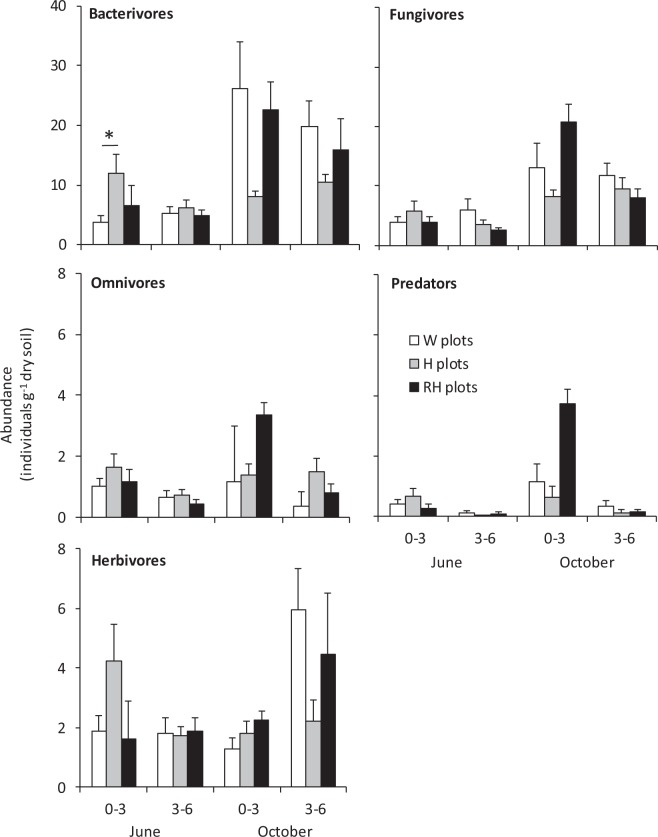
Abundance of nematode trophic groups (mean + s.e., n = 10) in the two layers of soil (0–3 and 3–6 cm) in field plots with non-treated weeds (W plots), hoeing (H plots) or Roundup application followed by hoeing (RH plots) in June and October samplings in 2016. Statistically significant differences of mean abundance in H plot–W plot and RH plot–H plot comparisons are depicted with a line and an asterisk (for the significance of soil layer and sampling month effects, see Table 1 and Supplementary Table 1).

The abundances of bacterivorous and fungivorous nematodes were affected by a significant soil layer × plot treatment interaction in the June 2016 sampling (Fig. 3, Table 1). For bacterivores, the interaction was because H and RH plots had higher abundances than W plots in the upper soil layer, but not in the lower layer (Fig. 3). For fungivores, no significant differences were found among plot treatment means in either soil layer despite the significant interaction effect (Fig. 3). Omnivores and predators were more abundant in the upper than lower soil layer in both samplings, whereas root feeders were more abundant in the lower layer in the October sampling (Fig. 3, Table 1). None of these trophic groups were affected by plot treatments (Fig. 3, Table 1).

All trophic groups, except for predators, were more abundant in October than June sampling in the lower soil layer, and the same trend was also found in the upper soil layer for bacterivores and fungivores (Fig. 3, Supplementary Table 1). The significant sampling month × plot treatment interaction effect on bacterivore numbers in the upper soil layer was due to significant treatments effects found in June, but not in October (Fig. 3, Supplementary Table 1).

**Figure 4:**
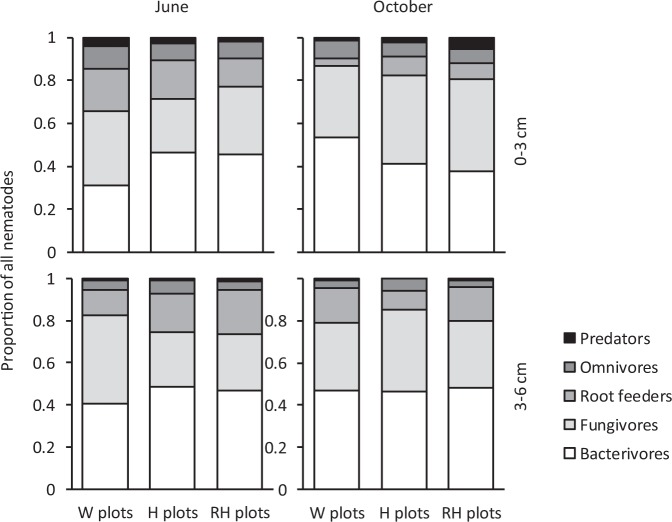
Relative abundance of nematode trophic groups (n = 10) in the two layers of soil (0–3 and 3–6 cm) in field plots with non-treated weeds (W plots), hoeing (H plots) or Roundup application followed by hoeing (RH plots) in June and October samplings in 2016 (for the significance of treatment, soil layer and sampling month effects, see Supplementary Table 2).

In the June 2016 sampling, bacterivores composed a higher and fungivores a lower proportion of total nematode community in H than W plots, while no differences were found between H and RH plots (Fig. 4, Supplementary Table 2a). For fungivores this pattern was found in both soil layers, whereas for bacterivores it was clear in the upper layer only (Fig. 4, Supplementary Table 2b). In the upper layer, root feeders made up a bigger proportion and fungivores a smaller proportion of the community in June than October (Fig. 4, Supplementary Table 2b). Omnivores and predators were relatively more abundant in the upper than lower layer in both samplings, and while this was also true for fungivores in October, root feeders showed an opposite pattern in October (Fig. 4, Supplementary Table 2a).

### Litter mass loss and resin capture of mineral N

**Figure 5:**
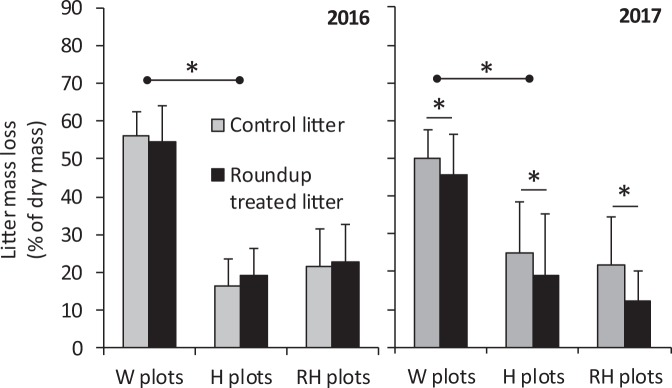
Mass loss (mean + s.e., n = 10) of litter sprayed with either water (control) or Roundup solution and placed on the ground in field plots with non-treated weeds (W plots), hoeing (H plots) or Roundup application followed by hoeing (RH plots) in 2016 and 2017. Statistically significant differences of mean mass loss in H plot–W plot, RH plot–H plot and control litter–Roundup treated litter comparisons are depicted with a line and an asterisk.

Litter mass loss was significantly higher in W than H plots, but did not differ between H and RH plots (Fig. 5, Table 2). Litter treated with Roundup solution had lower mass loss than litter treated with water in 2017, but not in 2016 (Fig. 5, Table 2).

**Table 2:** F and P statistics of repeated measures ANOVA of the effects of litter treatment (water or Roundup application, a repeated measure) and plot treatment [non-treated weeds (W), hoeing (H), Roundup application followed by hoeing (RH)] on litter mass loss (% of dry mass) in growing seasons 2016 and 2017, supplied with a *post hoc* SNK test of the statistical significance of differences among plot treatments.

	Litter treatment		Litter treatment × Plot treatment		Plot treatment		SNK test
	F	P	F	P	F	P	
2016	0.43	0.521	0.73	0.498	101	**<0.001**	W > H, H = RH
2017	5.05	**0.040**	0.21	0.812	24.8	**<0.001**	W > H, H = RH

Field replicate block was included in ANOVA models, but is not reported in the table. Statistically significant (P < 0.05) effects are marked in bold.

**Figure 6:**
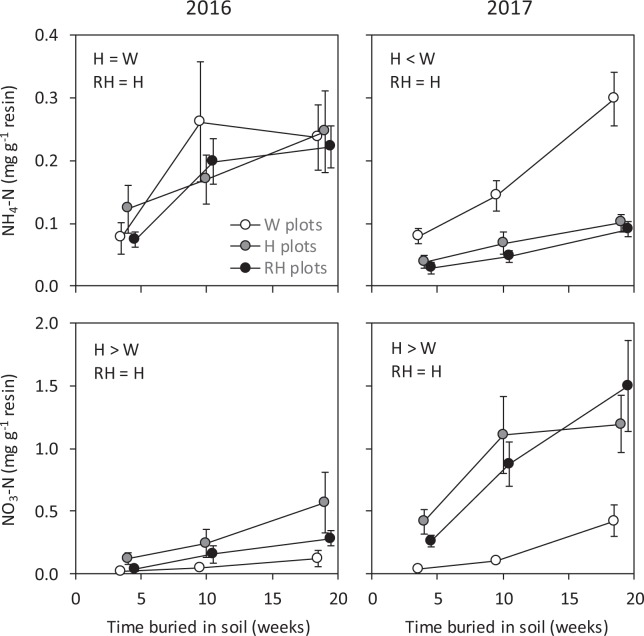
Amount of inorganic N (mean ± s.e., n = 10) captured by ion exchange resin capsules in 4, 10 and 19 weeks in the soil of field plots with non-treated weeds (W plots), hoeing (H plots) or Roundup application followed by hoeing (RH plots) in 2016 and 2017. The results of H plot–W plot and RH plot–H plot comparisons are given (for the significance of burial time effects, see Table 3).

The amount of NH_4_-N captured by resin capsules was not affected by plot treatments in 2016, but was lower in H than W plots in 2017 (Fig. 6, Table 3). The amount captured in H and RH plots did not differ in 2017, and the significant sampling × plot treatment interaction effect on NH_4_-N capture (Table 3) was due to an increasing difference between W plots and the two other treatments (Fig. 6). Resin NO_3_-N capture was higher in H than W plots in both years, but did not differ between H and RH plots (Fig. 6, Table 3).

**Table 3:** F and P statistics of repeated measures ANOVA of the effects of time being buried in soil (4, 10 and 19 weeks, a repeated measure) and plot treatment [non-treated weeds (W), hoeing (H), Roundup application followed by hoeing (RH)] on resin NH_4_- and NO_3_-N capture through growing seasons 2016 and 2017, supplied with a *post hoc* SNK test of the statistical significance of differences among plot treatments.

	Time being buried		Time × Plot treatment		Plot treatment		SNK test
	F	P	F	P	F	P	
**2016**							
Resin NH_4_-N capture	10.3	**<0.001**	0.75	0.565	0.23	0.795	
Resin NO_3_-N capture	9.64	**0.003**	1.49	0.247	3.69	**0.046**	W < H, H = RH
**2017**							
Resin NH_4_-N capture	26.9	**<0.001**	5.96	**0.004**	43.2	**<0.001**	W > H, H = HR^a^
Resin NO_3_-N capture	18.0	**<0.001**	2.31	0.077	10.22	**0.001**	W < H, H = RH

Field replicate block was included in ANOVA models, but is not reported in the table. Statistically significant (P < 0.05) effects are marked in bold.

^a^True for each burial time.

### Soil glyphosate concentrations

No glyphosate or AMPA remains were found in soil samples collected from RH and H plots at the end of the experiment.

## Discussion

We predicted that killing live vegetation will have clear impacts on the abundance of soil fauna, litter mass loss and soil mineral N availability. This prediction was fully confirmed in terms of ecosystem functioning: litter mass loss and resin NH_4_ capture were lower and resin NO_3_ capture higher in H than W plots. In soil fauna, the effects were more mixed with enchytraeids having no response and the response of nematodes depending on the soil layer and study year. Immediately after the first treatment session in June 2016, elevated numbers of nematodes, and especially bacterivorous nematodes, were found in the upper layer of H plots, but throughout the second study year, the effect of hoeing on nematode numbers was clearly negative in both soil layers. Our second prediction was that the effects of killing plants with a Roundup treatment before hoeing would not significantly differ from the effects of hoeing alone. This prediction was confirmed for the most part: animal abundances, litter mass loss and soil mineral N availability showed no differences between H and RH plots except for the number of total nematodes, which was lower in the upper soil layer in RH than H plots in the June 2016 sampling. Another indication of Roundup effects that we observed was a decreased mass loss of plant litter sprayed with Roundup in 2017. Taken together, our results suggest that recognizing the effects of killing plants as such is essential when testing direct herbicide effects on the structure and functioning of soil communities. These effects can be extensive and need to be carefully disentangled from direct herbicide effects. Secondly, our results suggest that treating live plants or plant litter with Roundup may not have major effects on the structure and functioning of soil decomposer communities.

Due to the high functional redundancy in species rich soil communities^43^, relations between soil biodiversity and ecosystem functioning typically depend more on the structural and functional diversity of soil organisms than on their species richness or other taxonomic parameters^19^. In our study, we therefore focused on the trophic groups of soil micro- and mesofauna rather than on their taxonomy. We used nematodes and enchytraeids as study organisms because their abundances are known to quickly respond to disturbances^36–39^, soil nutrient mineralization is controlled by these animals^15–18^ and changes in the abundance of bacterial- and fungal-feeding nematodes can be used as surrogates of changes in bacterial and fungal growth^40–42^. We expected that the different trophic groups would respond to hoeing as it affects the basal resource supply of soil food webs by killing live roots, terminating root carbon release and increasing dead plant material. Indeed, of the nematodes, the abundance and relative proportion of bacterial feeders was increased and the relative proportion of fungal feeders decreased by hoeing in the June 2016 sampling, suggesting that bacterial growth in particular was enhanced by greater plant litter supply in H plots. However, this positive effect was already absent in the October 2016 sampling and in 2017, the total number of nematodes was 75% lower in H than W plots, which illustrates the dependence of nematode abundance on continuous primary production. None of the trophic groups nor the community composition responded to the Roundup treatment in 2016, i.e. the abundances or relative proportions of trophic groups did not differ between H and RH plots, whereas the total number of nematodes was lower in RH than H plots in the June 2016 sampling. This effect is hard to explain as none of the trophic groups had a parallel pattern, and as this effect did not take place in any other sampling, its importance appears limited. Overall then, the effects of Roundup on nematode abundances can be considered negligible. We neither found effects of Roundup on the biomass of enchytraeids. While this was in line with our prediction, we expected that enchytraeids would respond to hoeing. Their numbers have been shown to respond to the availability of decomposing plant material^31^ and changes in soil density^39,44^, which both were likely affected by hoeing. It might be, however, that the effects of hoeing on the physical and nutritional conditions of enchytraeids did not significantly differ from those caused by the earlier tilling of the experimental field.

There is limited earlier evidence of glyphosate effects on soil nematodes, and no evidence of effects on enchytraeids, but Zhao *et al*.^45^. recently published a meta-analysis of nematode responses to different herbicides. Their analysis suggests that the abundance of bacterial-feeding, plant parasitic and omnivorous nematodes increase in herbicide treated soils, while those of fungivorous and predatory nematodes decrease. However, it is not clear whether the studies in the meta-analysis had distinguished the direct herbicide effects from the indirect effects mediated through reduced root carbon flow and increased dead plant material. Moreover, only one of the studies focused on glyphosate effects. In that three-year study, Liphadzi *et al*.^46^ showed that the total nematode abundance and the proportions of trophic groups in conventionally tilled and no-till soybean and corn fields in Kansas, USA, did not under glyphosate application (1.12 kg ha^−1^ applied once or twice during the growth period) differ from those observed under application of other herbicides. To our knowledge, this study is the only field experiment of glyphosate effects on soil nematodes and since then, only one short-term laboratory experiment of the effects of single glyphosate application on the nematodes of Australian banana plantation soil has been published^47^. In this study, no significant effects of glyphosate application on the total number of nematodes or nematode trophic groups were found. Our results are in line with these findings and suggest that major glyphosate effects on soil nematodes are neither likely in soils under cold climatic conditions. We did not examine the whole decomposer food web, and it is possible that other groups of organisms such as protozoa and arthropods are more sensitive to Roundup than nematodes and enchytraeids. However, considering that our target organisms covered all trophic groups of soil animals and included microbial feeders and their predators, major changes within the decomposer food web should have become visible through competitive and predatory interactions. It also appears that our sampling protocol was efficient enough to notice changes in nematode and enchytraeid abundances as manifested by the observed differences between soil layers, seasons and the W and H plots.

To examine the effect of Roundup on soil ecosystem services supplied by decomposer organisms, we measured plant litter mass loss and soil availability of mineral N. As we hypothesized, destroying live vegetation affected litter mass loss. The lower mass loss in H than W plots can be explained by litter being on average drier and microbial activity therefore lower on the exposed soil surface than among dense vegetation. The higher soil NO_3_^−^ availability in H than W plots was in turn most likely a consequence of two processes: first, there was more dead plant material (i.e. organic N) available for microbial consumption and N mineralization in H than W plots, and secondly, plants did not exploit NO_3_^−^ in H plots. In 2017, more NH_4_^+^ was found in W than H plots. This was unexpected since plants utilize both mineral forms of N and their concentrations in soil should therefore be lower under active vegetation than in bare soil. The explanation is therefore likely related to differences in the activity of nitrification bacteria between W and H plots. We did not measure soil temperature, but most likely the soil was on average warmer in open, hoed plots than in shaded weed plots and the warmer soil may have raised the nitrification rate (i.e. the microbial oxidation of NH_4_^+^ to NO_3_^−^) in the hoed plots. Overall, in contrast to these clear effects of plant absence on soil functioning, no differences in functioning were detected between H and RH plots. This suggests that killing weeds using Roundup, even when applying the highest allowable dose, may not have impacts on soil functioning.

The only indication of direct Roundup effects on soil functioning that we found was a 20% decrease in the mass loss of litter sprayed with Roundup in 2017. This finding suggests that in places where plant remains are subjected to herbicide application after crop plant harvest, herbicide effects on decomposers may also be transmitted by plant litter. The effect was most likely not due to Roundup-induced changes in litter C/N-ratio^48^ as the slightly increased N concentration and reduced C/N –ratio should have enhanced rather than reduced microbial growth. Hoesel *et al*.^49^ recently found no effects of Roundup application on decomposition using a tea bag method, but no studies have to date examined the decomposition rate of glyphosate treated plant litter. Most of the studies that have tested the impact of Roundup on soil micro-organisms indicate negligible or minor effects on microbial community structure when the herbicide is applied in recommended doses^50–53^. Increased microbial activity has also been reported, which indicates that microbes can utilize Roundup as a source of carbon, nitrogen or phosphorus^52,54–56^. In our study, the litter was only accessible to soil microbes and micro- and mesofauna, not to macrofauna such as earthworms, whose straw incorporation activity has been reported to decrease after glyphosate spraying^29,48^. The reduced litter mass loss we observed therefore needs to be due to changes in the activity of microbes and/or the micro- and mesofauna. The reason for decreased litter degradation in 2017, but not in 2016, could be related to higher precipitation and higher overall microbial activity in summer 2017, but this idea is not supported by equal mean litter mass loss in 2016 and 2017. All in all, considering that reduced litter mass loss was observed in one year only and that the effect was statistically not highly significant (thus having relatively high possibility of being coincidental), further field-scale tests of the effects of subjecting plant litter to Roundup are warranted. It is also important to note that glyphosate was in our study applied as a formulated product, Roundup Gold, which contains a surfactant, etheralkylamine ethoxylate (EtO-EA: CAS 68478-96-6). There is evidence that EtO-EA is toxic to human cells^57,58^, but we are not aware of any studies that would have tested its effects on soil microbes. Nevertheless, when carrying out further tests of glyphosate risks, this is a viewpoint that might be worth of more attention.

In a recent analysis of 300 top-soil samples collected in EU, Silva *et al*.^59^ showed that 21% of the soils had glyphosate concentrations and 42% of soils AMPA concentrations higher than 0.05 mg kg^−1^, while only few exceeded the maximum glyphosate Predicted Environmental Concentration PEC value (for cereals) of 0.30 mg kg^−1^ ^60^. In Finland, Laitinen *et al*.^8^ have reported glyphosate concentrations of 0.35 mg kg^−1^ in agricultural soils. Although we found no glyphosate or AMPA remains in our study at the end of the growing season 2017, glyphosate concentrations of 0.3–0.5 mg kg^−1^ soil were recorded in early summer samples (collected ca. 8 months after autumn sprayings) of another experiment in our field site (unpublished data). In this experiment, treatment plots were sprayed simultaneously with our plots using the same Roundup solution, spraying equipment and field personnel as in our study. Also in this experiment, however, no remains were found in Roundup treated soils at the end of the growing season 2017, which supports our conclusion that glyphosate was properly degraded in our experiment. In the soil, glyphosate degradation is mainly a microbiological process and degradation times (DT_50_) can vary from a few days to several months, with some observations of degradation lasting for years^59^. It has been argued that the short biologically active season could restrict the degradation of glyphosate in northern countries and special attention should therefore be paid on glyphosate application^9^. Our results suggest, however, that glyphosate and AMPA remains can be effectively degraded also in northern clay soils under their typical climatic conditions.

## Conclusions

Compared with the increasing public fear of the risks related to the massive, worldwide use of glyphosate-based herbicides in agriculture, their field-scale, long-term tests in agricultural soils have been rare. To our knowledge, our study is the first test of undesirable glyphosate effects in agriculture that combines several soil trophic groups with the two main soil ecosystem services in a well-replicated, properly controlled field study. Our results give rise to three conclusions, one for testing glyphosate effects and two for applying Roundup in agriculture. First, terminating primary production by killing plants has drastic effects on soil heterotrophic organisms and the processes they govern. Distinguishing these effects from direct glyphosate effects is therefore profoundly important when evaluating glyphosate risks in soils. Second, it appears that the glyphosate that enters the soil can be quickly degraded also under northern climatic conditions. Third, when glyphosate degradation is effective and Roundup is used within recommended limits, the effect of weed control with Roundup likely has minor and transient effects on the structure and functioning of food webs in agricultural soils.

## Materials and Methods

### Field site

The experiment was carried out in an agricultural field (50 m × 25 m) in the Ruissalo Botanical Garden of the University of Turku in south-west Finland (60.4333°N, 22.1733°E). Air temperatures in the years of the experiment (2016 and 2017) were typical to the region (Finnish Meteorological Institute, 2018): the mean annual temperatures were 6.4 and 6.5 °C and the mean summer (May–July) temperatures 16.6 and 15.5 °C, respectively. Year 2016 was dry, with precipitation reaching 495 mm only, while in 2017, precipitation was 652 mm, near to the 30-year mean annual precipitation of 687 mm in Ruissalo. During summer months (May–August), precipitation was ca. 170 mm in both years, which remains below the 30-year mean of 211 mm. When the field was established in 2013, the suitability of soil for crop cultivation was enhanced by peat (12 m^3^) and sand (12 m^3^) addition and the soil was tilled to a depth of 15 cm. In 2013–2015, the field was tilled twice a year (May and October) to a depth of 5 cm using a hand rotary tiller and cultivated with barley and potato. Based on the USDA soil texture classification, the soil in the field is loamy clay with high organic matter content (after peat addition >120 g kg^−1^). When the current experiment was established in May 2016, soil pH was measured using 1:2.5 (V/V) soil:distilled water suspension (ISO 10390 standard), nutritional status (Ca, K, Mg, P) was determined according to Vuorinen and Mäkitie^61^, and soil C and N concentrations were measured using a LECO CNS-2000 analyzer (Leco Corporation, USA). The values of these soil attributes are presented in Table 4. The field site had no previous history of herbicide application.

**Table 4:** Nutrient concentrations and other characteristics of the experimental field soil (n = 3; CEC = Cation exchange capacity, EC = Electrical conductivity).

		Unit	Mean	SE
Soluble	Ca	mg/kg	894	47
	P	mg/kg	7	0.7
	K	mg/kg	395	17
	Mg	mg/kg	645	61
	S	mg/kg	27	2
	Cu	mg/kg	11	0.1
	Mn	mg/kg	21	3
	Zn	mg/kg	4	0.1
Total	Ca	mg/kg	2871	263
	K	mg/kg	4280	79
	Mg	mg/kg	6638	1081
	P	mg/kg	364	25
	CEC	mmol/kg	17	0.3
	Ca/CEC	%	47	0.9
	K/CEC	%	4	0.3
	Mg/CEC	%	22	1.9
	Na/CEC	%	2	0.3
	EC	mS/cm	7	0.7
	pH		5.9	0.09

### Experimental set-up and treatments

When the experiment was started in May 2016, the field was fully covered by “weeds” such as *Elymus repens*, *Trifolium pratense*, *Ranunculus repens* and *Taraxacum officinale*. Thirty experimental plots (1.0 m × 1.5 m) were established in ten replicate blocks and the three treatments were randomly allocated to plots in each block. The distance among plots within the block was 0.5 m, while the blocks were 2 m apart. Each treatment plot was either (1) sprayed with 1 l of tap water (a non-treated weed, or W plot), (2) hoed to a depth of 3 cm using Fiskars Quikfit 1000738 hoes and sprayed with 1 l of tap water (a hoed, or H plot), or (3) sprayed with Roundup Gold (glyphosate concentration 450 g l^−1^, CAS: 38641-94-0, application rate 6.4 l ha^−1^ in 1 l of tap water per plot) and later hoed (a RH plot). As effective control of perennial weeds requires high doses and we wanted information of the highest, but still realistic risk in agricultural use, we sprayed the plants with the maximal permitted glyphosate dosage (3 kg ha^−1^) using hand-operated pressure tank with a manual sprayer. The plots were treated twice a year, on 23th of May and 3rd of September, in both 2016 and 2017. First hoeing was carried out in H plots in the same day as the glyphosate application in RH plots and repeated thrice during the next 11 d. After 11 d, when the plants had withered both in the RH and H plots, the RH pots were hoed thrice to a depth of 3 cm to achieve similar soil structure and disturbance as in H plots. The plots were not tilled during the experiment.

### Abundance of soil animals and soil moisture

To analyze the effects of treatments on the abundance of nematodes (representing the microfauna) and enchytraeids (representing the mesofauna), three randomly placed soil cores (depth 6 cm, diameter 3 cm) were collected from each plot on 20th of June (i.e. 4 weeks after spring treatment) and 10th of October (i.e. 4 weeks after autumn treatment) in both 2016 and 2017. The soil cores were cut horizontally in half to distinguish animal responses in the top (0–3 cm) and lower (3–6 cm) soil layer. These layers were analyzed separately, but for each layer, the three soil cores collected from the same plot were pooled to attain one estimate per plot. Nematodes were extracted from ca. 8 g and enchytraeids from ca. 80 g of fresh, non-sieved soil using the wet funnel methods by Sohlenius^62^ and O’Connor^63^, respectively. The number of nematodes was counted and their feeding, or trophic groups were identified^21^. In 2017, nematode numbers were so low in some treatments that estimates of trophic group abundances were not reliable and the results are presented as a total number only. To further examine the effects of treatments on the community composition of nematodes, relative proportions of trophic groups were calculated for 2016. Enchytraeids were counted and classified into size classes (length 0–2, 2.1–4, 4.1–6, 6.1–8, 8.1–10, 10.1–12 or >12 mm) and their biomass was calculated according to Abrahamsen^64^. Nematode and enchytraeid abundances are expressed per g of soil dry matter. The water content of soil samples was determined by weighing subsamples before and after drying in an oven (105 °C) for 24 h.

### Litter mass loss and soil mineral N availability

Effects of plot treatments on ecosystem services were assessed through changes in litter mass loss and soil availability of mineral N, i.e. NO_3_^−^ and NH_4_^+^. Litter mass loss was assessed using litter bags of overwintered, erect meadow fescue (*Festuca pratensis*) shoot litter, including both senescent leaves and stems. The litter was collected from grassland, air dried, cut into 2 cm pieces and stored at room temperature until 2 g of litter (dry mass equivalent) was placed into mesh bags (7 × 7 cm, mesh size 0.1 mm). The litter placed into mesh bags was not oven dried to preserve microbes living on senescent plant parts, but instead subsamples of litter were dried (48 h, 70 °C) and their water content determined to calculate the dry mass of the added litter. To test the consequences of exposing dead plant remains to a Roundup treatment, one half of the bags was filled with litter sprayed with water and the other half with litter sprayed with Roundup Gold solution. The applied dose for the litter treatment was chosen to represent the maximum allowed deposit in agricultural fields (i.e. 0.64 ml herbicide per 6 l dry litter, which was estimated to correspond to a volume of plant remains per square meter after crop harvesting). After treatment, the herbicide treated litter had a C concentration of 44.25%, a N concentration of 1.24% and a C/N–ratio of 35.9, while the corresponding values for water treated litter were 44.21%, 1.21% and 36.5, respectively (analyzed using a LECO CNS-2000 analyzer). One bag of each type was then placed on the soil surface in each field plot 11 d after starting the field treatments (3rd of June in both 2016 and 2017). The bags were covered using a piece of grey mesh and collected on 10th of October in the year of placement. The collected bags were stored frozen (−18 °C) until litter was dried (48 h, 70 °C) and weighed. As the mesh size was small and the bags were placed on the soil surface, no soil was assumed to enter the bags and was thus not quantified.

The availability of NH_4_^+^ and NO_3_^−^ in the soil was estimated using capsules filled with 1 g of ion exchange resin (UNIBEST Ag Manager™). Three capsules were buried in the depth of 3 cm in each plot on 3rd of June in both 2016 and 2017. One capsule was then collected after 4, 10 and 19 weeks from each plot and stored at 4 °C until processed. In the laboratory, the capsules were rinsed with distilled water and their ion content extracted using 50 ml of 2 M KCl. The obtained solutions were filtered through glass microfiber filters (Whatman GF/C) and their NH_4_^+^ and NO_3_^−^ contents analyzed using a Lachat QuickChem 800 Analyzer (Zallweger Analytics, Inc., Lachat Instruments Division, USA).

### Soil glyphosate concentrations

The glyphosate and AMPA concentrations in soil samples collected from Roundup treated (RH) and hoed (H) plots at the end of the study in October 2017 were analyzed in Groen Agro Control (www.agrocontrol.nl). Due to the high cost of glyphosate analysis, samples collected from H and RH plots were randomly pooled to achieve two composite samples for both H and RH plots.

### Statistical analyses

The effects of plot treatment (W, H and RH) on soil moisture, abundances of soil fauna (including the abundance and relative proportions of nematode trophic groups), litter mass loss and resin N capture were tested using ANOVA models for repeated measures, where the plot treatment was treated as a fixed factor and the repeated measures were comprised of either soil layers (vertically repeated measure of plot treatment effects), samplings (temporally repeated measure of plot treatment effects) or treatments of decomposing litter (experimentally repeated measure of plot treatment effects). While measures of soil moisture, litter mass loss and resin N capture were repeated in one dimension only, measures of soil fauna were repeated both vertically and temporally. Therefore, to be able to test whether plot treatment effects on soil fauna depended on soil layer or sampling month (i.e. whether significant soil layer × plot treatment or sampling month × plot treatment interaction effects appeared), two separate models were used with the soil layer (Table 1, Supplementary Table 2a) and sampling month (Supplementary Table 1, Supplementary Table 2b) in turn as a repeated measure. Once statistically significant plot treatment effects appeared in ANOVA models, the statistical significance of differences between W and H plots and between H and RH plots were interpreted using a Student-Newman-Keuls (SNK) *post hoc* test, a powerful test suitable for comparing three means^65^. The replicate block was included in all models to explain spatial variation within the experimental field, and the homogeneity of variances and normal distribution of model residuals were tested using Levene’s test and Shapiro-Wilk test, respectively. To fulfill the assumptions of ANOVA, all abundances of soil fauna were log-transformed. All statistical analyses were carried out using the SPSS statistical package (IBM Corp. 2016).

## Supplementary information

Supplementary material

## Data Availability

The datasets generated and/or analysed during the current study are available from corresponding author on a reasonable request.
