# Supplementary material for: Effects of a glyphosate-based herbicide on soil animal trophic groups and associated ecosystem functioning in a northern agricultural field
Source: Sci Rep. 2019 Jun 12;9:8540. doi: 10.1038/s41598-019-44988-5 (PMC6561955; doi:10.1038/s41598-019-44988-5)
Supplement: Supplementary file 1 — Supplementary material [file 41598_2019_44988_MOESM1_ESM.pdf]

Effects of a glyphosate-based herbicide on soil animal trophic groups and associated ecosystem functioning in a northern agricultural field

Marleena Hagner, Juha Mikola, Irma Saloniemi, Kari Saikkonen and Marjo Helander

Supplementary Table 1. F and P statistics of repeated measures ANOVA of the effects of sampling month (June, October; a repeated measure) and plot treatment (non-treated weeds, hoeing, Roundup application followed by hoeing) on nematode numbers and enchytraeid biomass in the two layers of soil (upper 0-3 cm, lower 3-6 cm) in growing seasons 2016 and 2017. Field replicate block was included in ANOVA models, but is not reported in the table. Statistically significant (P < 0.05) effects are marked in bold.

	Sampling month		Month × Plot treatment		Plot treatment	
	F	P	F	P	F	P
**2016, upper layer**						
Enchytraeid biomass	0.11	0.746	2.71	0.093	1.22	0.319
Total nematode number	6.85	**0.017**	4.25	**0.031**	0.39	0.686
Bacterivores	7.40	**0.014**	6.78	**0.006**	0.50	0.613
Fungivores	20.4	**<0.001**	0.78	0.472	0.03	0.973
Root feeders	3.25	0.088	0.38	0.689	1.99	0.166
Omnivores	1.38	0.255	0.93	0.414	0.32	0.733
Predators	2.81	0.111	1.97	0.168	0.39	0.682
**2016, lower layer**						
Enchytraeid biomass	2.66	0.120	0.63	0.543	1.47	0.257
Total nematode number	45.9	**<0.001**	0.35	0.713	0.96	0.402
Bacterivores	31.1	**<0.001**	1.06	0.367	0.35	0.713
Fungivores	58.8	**<0.001**	0.15	0.865	2.95	0.078
Root feeders	5.52	**0.030**	1.95	0.172	1.04	0.373
Omnivores	9.24	**0.007**	0.23	0.798	1.42	0.267
Predators	2.11	0.164	0.38	0.688	2.36	0.123
**2017, upper layer**						
Enchytraeid biomass	2.08	0.166	1.58	0.233	1.79	0.196
Total nematode number	2.34	0.143	1.36	0.282	10.5	**0.001**
**2017, lower layer**						
Enchytraeid biomass	0.50	0.489	0.57	0.576	0.09	0.917
Total nematode number	4.02	0.060	2.67	0.097	18.7	**<0.001**

Supplementary Table 2. F and P statistics of repeated measures ANOVA of the effects of (a) soil layer (upper 0-3 cm, lower 3-6 cm; a repeated measure) and plot treatment (non-treated weeds, hoeing, Roundup application followed by hoeing) and (b) sampling month (June, October; a repeated measure) and plot treatment on the relative proportions of nematode trophic groups of the total nematode number in 2016. Field replicate block was included in ANOVA models, but is not reported in the table. Statistically significant ($P < 0.05$) effects are marked in bold.

(a)	Soil layer		Soil layer × Plot treatment		Plot treatment	
	F	P	F	P	F	P
**June**						
Bacterivores	2.71	0.117	1.08	0.361	4.82	**0.021**
Fungivores	0.21	0.655	1.36	0.282	7.36	**0.005**
Root feeders	<0.01	0.945	2.43	0.117	0.16	0.854
Omnivores	5.61	**0.029**	0.74	0.491	1.06	0.369
Predators	8.30	**0.010**	1.58	0.232	0.76	0.484
**October**						
Bacterivores	1.25	0.278	3.25	0.062	1.46	0.259
Fungivores	6.02	**0.025**	2.51	0.109	1.35	0.284
Root feeders	7.95	**0.011**	2.27	0.132	0.37	0.693
Omnivores	5.25	**0.034**	0.70	0.511	0.23	0.795
Predators	10.1	**0.005**	2.26	0.134	2.47	0.113

(b)	Month		Month × Plot treatment		Plot treatment	
	F	P	F	P	F	P
**Upper soil layer**						
Bacterivores	0.84	0.372	8.81	**0.002**	0.10	0.903
Fungivores	11.2	**0.004**	3.93	**0.038**	0.64	0.539
Root feeders	27.4	**<0.001**	2.05	0.158	0.44	0.652
Omnivores	0.91	0.354	0.10	0.906	1.27	0.304
Predators	<0.01	0.948	3.09	0.070	0.40	0.679
**Lower soil layer**						
Bacterivores	0.57	0.461	0.91	0.419	0.79	0.471
Fungivores	0.69	0.419	4.30	**0.030**	3.15	0.067
Root feeders	1.96	0.178	2.42	0.117	0.72	0.499
Omnivores	1.09	0.309	0.02	0.978	0.92	0.416
Predators	0.37	0.554	0.52	0.604	1.22	0.317
